# Serum vitamin D and PSA in elderly men in Amirkola

**DOI:** 10.22088/cjim.15.3.535

**Published:** 2024-08-01

**Authors:** Parvin Sajadi Kaboudi, Maryam Halakoo, Khadijeh Ezoji, Hamid Shafee, Seyed Reza Hosseini, Ali Bijani

**Affiliations:** 1 Social Determinants of Health Research Center, Health Research Institute, Babol University of Medical Sciences, Babol, Iran; 2 Student Research Committee, Babol University of Medical Sciences, Babol, I.R. Iran; 3 Infertility and Reproductive Health Research Center, Health Research Institute, Babol University of Medical Sciences, Babol, Iran

**Keywords:** Serum PSA level, Vitamin D, Elderly

## Abstract

**Background:**

Vitamin D is a modifiable risk factor in cancer and prostate diseases. In this study, we investigate the relationship between vitamin D and serum PSA in elderly men of Amirkola City.

**Methods:**

The current cross-sectional descriptive study was conducted on elderly men participating in the cohort study in Amirkola. Demographic information including age, sex, marital status and occupation were recorded and blood samples (5 cc of blood) were taken to measure PSA and vitamin D. A p -value less than 0.05 is statistically significant.

**Results:**

After applying the inclusion and exclusion criteria, 837 elderly men with mean age of 69.99 ***±*** 7.72 years were included in the study. In terms of marital status, 779 (93.1%) were married and 59 (6.9%) were single. In the study of employment status, 476 (56.9%) self-employed, 331 (439.5%) retired, 8 (1.0 %) housewives, 14 (1.7%) unemployed and 8 (1.0 %) They were in an unknown situation. The mean level of vitamin D was 31.94 ***±*** 28.57 ng / mL and the mean level of PSA was 1.94 ± 3.28 ng / dL. No significant relationship was found between vitamin D level and serum PSA in Pearson Correlation test (P = 0.16). Among the other variables studied, only age was related to PSA levels and PSA level increased with age (P = 0.001).

**Conclusion:**

No significant relationship was found between PSA serum level and vitamin D level, but the existence of vitamin D deficiency in most of the elderly studied needs attention.

During the last half century, the number of people aged 60 and over has increased by an average of 8 million people per year worldwide; Our country is not an exception to this rule and statistical indicators show that the aging process has started in our country as well (1). Aging is associated with many diseases, including prostate cancer, which is the second most common cancer in men and the fifth cause of death due to cancer(2). The causes of prostate cancer can be divided into two ,non-modifiable groups, including old age, family history, socioeconomic status, race, and modifiable, including access to medical care, obesity, physical activity, and diet (2, 3). Recent studies have focused on 25(OH)vit D as a modifiable and effective factor in prostate cancer and we know more than 90% of this vitamin is supplied through the ultraviolet rays of the sun and it is achievable (3). The role of vitamin D in reducing the risk of prostate cancer is assumed based on the observation of a higher death rate due to this cancer in areas with less sunlight and a higher prevalence of this cancer in African men, northern latitudes and old age, because all these conditions are associated with decreased serum levels of vitamin D are associated (4). Vitamin D prevents cell proliferation and causes cell differentiation and apoptosis (5-7). It also reduces prostate tumor growth and inhibits its metastasis in rodents (8, 9). In some studies, the relationship between vitamin D level and prostate volume has been stated even in patients with Benign Prostate Hyperplasia (BPH) (10, 11). In fact, PSA is one of the proteins on the surface of prostate gland cells, whose serum level increases in cases of malignancy, inflammation or prostate hyperplasia (3).

Considering that 25(OH) vit D is a modifiable risk factor in prostate diseases and the common deficiency of this vitamin in the country, in this study we decided to examine the relationship between vitamin D and serum PSA in elderly men of Amirkola City.

## Methods

The present cross-sectional study was conducted on elderly men who were in the first phase of Amirkola elderly cohort study (AHAP) (12). In this cohort study, after obtaining consent, demographic information including age, body mass index, marital status and occupation, as well as blood sampling (5 cc of blood) were taken to measure PSA and vitamin D. Blood samples of the elderly were sent to the laboratory of the Cellular and Molecular Research Center of Babol University of Medical Sciences to measure vitamin D levels and PSA levels. In this study, based on the vitamin D levels of the elderly in three groups, normal vitamin D level (more than 30 ng/ml), insufficient vitamin D level (equal to 20 to 30 ng/ml) and vitamin D deficiency (less than 20 ng/ml) were divided and the active form of vitamin D was measured via the Diasorin kit using a quantitative luminescence method. The inclusion criteria in this study were all elderly men (60 years and older) living in Amir Kola, and the exclusion criteria were those with prostatitis, UTI, and BPH, and the use of vitamin D and calcium supplements, chronic kidney, liver, or intestinal diseases. Finally, 837 people were included in the study. In terms of body mass index, patients were also divided into three groups: BMI less than 25, BMI between 25 and 29.9 and BMI above 30. The normal range of PSA was considered to be less than 2.5 ng/dL. The ethical code of this study is IR.MUBABOL.REC.1399.244.

## Results

This cross-sectional study was conducted with the aim of investigating the relationship between PSA and VIT-D serum levels in 837 elderly men from Amir Kola, whose average age was 69.99±7.72 years.Age distribution of the participants in the study, 280 (33.5%) individuals between 60 and 64 years old, 163 (19.5%) 65 and 69 years old, 150 (17.9%) 70 and 74 years old, 138 (16.5%) 75 to 79 years old, 64 (7.6%) 80 and 84 years old, 42 (5%) individuals over 85 years old. In terms of marital status, 779 (93.1%) subjects were married and 58 (6.9%) were single (spouse died or divorced).

In the examination of the employment status, 476 (56.9%) people are employed other than housekeeping, 331 (39.5%) retired, 8(1%) housekeeping, 14(1.7%) unemployed, and 8 (1%) had an uncertain status. In the study of the level of education, 508 (60.7%) people were illiterate, 238 (28.4%) had primary and secondary education, and 91 (10.9%) with high school and university education. The patients were divided into three groups in terms of body mass index, 329 (40.8%) had a BMI less than 25, 354 (43.9%) with BMI between 25 and 29.9, and 124 (15.4%) between 25 and 29.9. They had a BMI higher than 30 (table 1).

**Table 1:** Demographic information of the studied elderly man of Amirkola City

Variable	Categories	**N (%)**
**Age **	60 to 64 years	280 (33.5%)
	65 to 69 years	163 (19.5%)
	70 to 74 years	150 (17.9%)
	75 to 79 years	138 (16.5%)
	80 to 84 years	64 (7.6%)
	Above 85 years	42 (5%)
**Marital status **	married	779 (93.1%)
	Single	58 (6.9)
**Employment status**	employed	476 (56.9%)
	Retired	331 (39.5%)
	housekeeping	8(1%)
	Unemployed	14(1.7%)
	Unknown	8(1%)
**BMI**	Less than 25	329 (40.8%)
	Between 25 and 29.9	354 (43.9%)
	Above 30	124 (15.4%)

**Figure 1:**
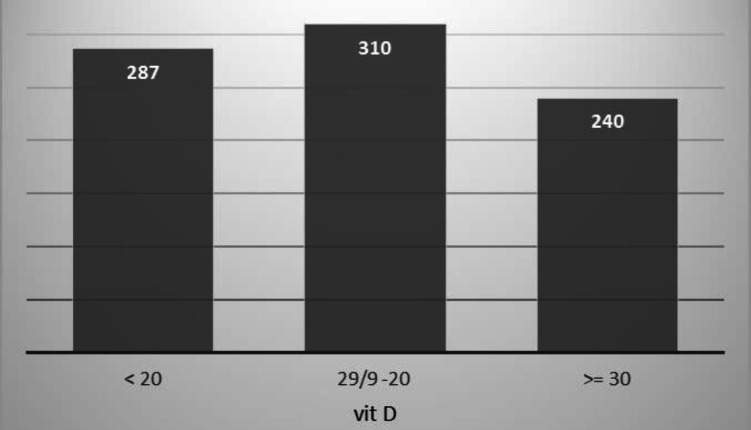
Frequency of vitamin D levels in the studied elderly men

In the examination of vitamin D level, 287 (34.3%) individuals with vitamin D level less than 20, 310 (37%) with vitamin D level between 20 and 29.9 and 240 (28.7%) with vitamin D level above 30 (Figure 1).

**Figure 2:**
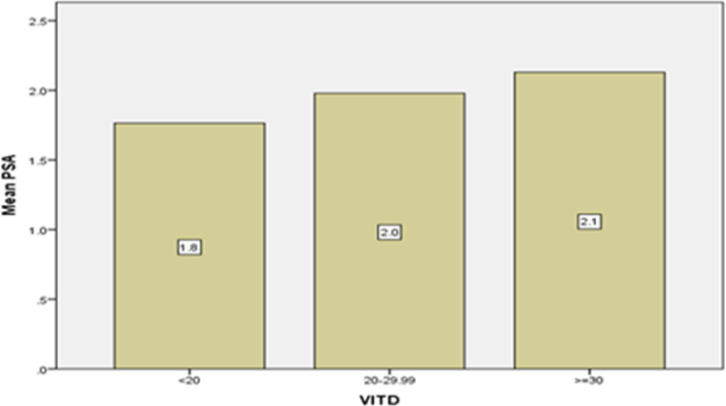
Average serum PSA level in elderly men of Amirkola City with different levels of vitamin D

**Figure 3:**
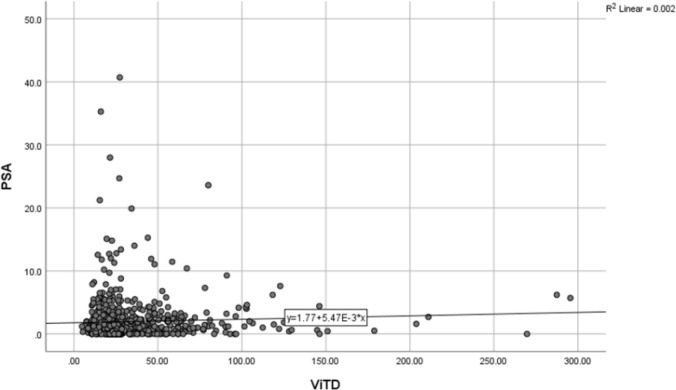
Distribution chart related to PSA levels in different amounts of vitamin D in the studied elderly

The mean PSA serum level in patients with vitamin D is less than 20 times 1.76±3.12 ng/dL while those with vitamin D levels is between 20 and 29.9 times 1.97±3.61 ng/dL and those with the level of vitamin D higher than 30 times, was 2.13±3.00 ng/dL found and that there was no significant difference between the above three groups in terms of PSA serum level (P=0.43) (Figure 2).The average vitamin D level was 31.94±28.57 ng/mL and the average PSA level was 1.94±3.28 ng/dL. In the examination of vitamin D level and PSA serum level, no significant relationship was found in the Pearson Correlation test with a correlation coefficient of 0.048 (P = 0.169) (Figure 3). 

**Figure 4:**
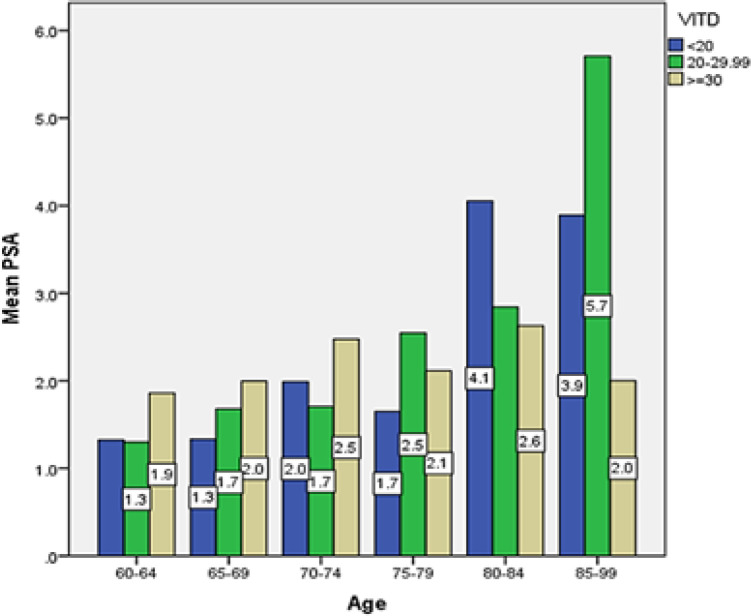
Average PSA serum level in elderly men of Amirkola City with different levels of vitamin D by age

In the study of age groups and PSA serum levels at different levels of vitamin D in the current study, the lowest average PSA serum level was found in the age group of 60 to 64 years and the highest in the age group above 85 years, where vitamin D level is between 20 and 30 had (1.3 and 5.7 ng/dL respectively). In the present study, a significant correlation was found between PSA serum level and age (P<0.001) (Figure 4). 

**Figure 5:**
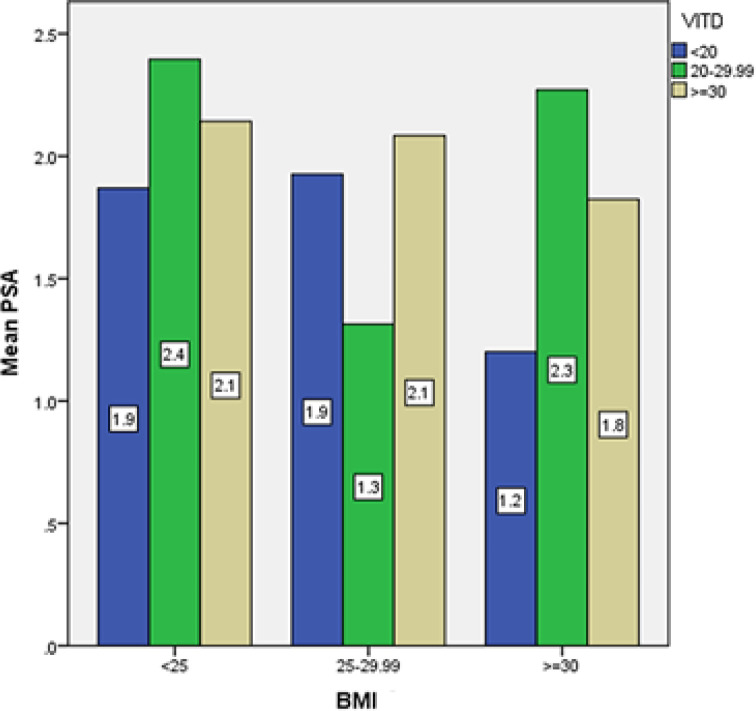
Average serum PSA in elderly men of Amirkola City with different levels of vitamin D in different BMI groups

In the study of different groups in terms of BMI, the lowest PSA serum level was found in the elderly with a BMI of more than 30 kg/m, whose vitamin D level was less than 20 (1.2 ng/dL), and the highest level was found in the elderly with a BMI of less than 25. It was found that their vitamin D level was between 20 and 30 (2.4 ng/dL). No significant relationship was found between PSA serum levels and vitamin D level according to BMI in the elderly (Figure 5). 

Regarding other variables of the study, no significant relationship was found between PSA levels and different levels of vitamin D (table 2). 

**Linear and multivariate regression analysis report** **: **In the regression analysis, no significant relationship was found between vitamin D level and serum PSA level, and among the other investigated variables, only age was related to PSA levels, which increases by one unit in the age score, the PSA level will increase by 3.71 (P=0.001) (table 3). 

**Table 2:** Average PSA levels in the study of some study variables in different levels of vitamin D in the studied elderly

**Vitamin D levels** **Variable**		**Above 30**	**20 to 30**	**Less than 20**	**P-value**
**Marital status**	**Single**	3.50±8.54	2.41±3.53	3.49±5.62	0.77
	**married**	1.66 ± 2.46	1.93±3.62	2.04 ± 2.76	0.33
**Employment status**	**housekeeping**	2/1	1.01±0.98	2.26 ± 2.87	0.71
	**Worker other than housekeeping**	1.57±1.80	1.85±2.90	2.31±3.45	0.06
	**Retired**	2.07±4.44	2.21 ± 4.47	1.87±2.23	0.82
	**Unemployed**	1.60 ± 1.78	0.90±0.39	1.40±1.06	0.78

**Table 3:** The results of multivariate regression analysis to predict serum PSA levels in elderly men of Amirkola City

**Predictor variables**	**B**	**SE**	**Beta**	**T**	**P-value**
**Vitamin D**	0.005	0.004	0.045	-1/26	0.207
**Age**	0.058	0.016	0.148	71/3	0.001
**Body mass index**	-0.005	0.027	-0.006	-0.17	0.86
**Employment status**	0.016	0.175	0.003	0.09	0.92

## Discussion

In the present study, the average vitamin D level was 31.94±28.57 ng/ml and the average PSA level was 1.94±3.28 ng/dL. In the examination of vitamin D level and PSA serum level, no significant relationship was found (P=0.16). In a study conducted in 2021 by Kumawat et al. on vitamin D deficiency and prostate cancer, the level of vitamin D in patients with prostate cancer was 15.71 ng/dL and 79.24% of patients with severe deficiency. Vitamin D had a PSA level above 20 ng/ml, and no significant relationship between vitamin D level and the risk of prostate cancer was found, which is consistent with the findings of the present study (13). 

In another study conducted by Toth et al., 5136 men were studied, the prevalence of vitamin D deficiency was found to be moderate in 55.8% and severe in 22.2%; In the above research, 22.3%, 18.2% and 23.4% of people with vitamin D levels below 15, 15 to 30 and above 30 micrograms per liter were people over 70 years old And in this study, a significant relationship was found between age and PSA level, but no significant relationship was found in the study of the relationship between PSA level and vitamin D serum level (14). 

Toprak B et al investigated the relationship between serum PSA concentration and vitamin D and total oxidant and antioxidant levels, and there was a significant relationship between serum PSA and total serum oxidant status (P=0.003), but there was a significant relationship between PSA and vitamin D levels. It was not found (P=0.23), which was consistent with the results found in most of the studies conducted in this field (15).

 In a study conducted by Chandler PD and colleagues on 105 healthy black men, they investigated the relationship between serum PSA levels and vitamin D; In the above research, which was conducted during three years and in the winter season, patients were examined in four groups (placebo, 1000 units daily, 2000 units daily and 4000 units of vitamin D daily) and the level of vitamin D before receiving the supplement and At the end of three months, it was measured that no relationship was found between serum PSA level and vitamin D in any of the studied groups, and it was consistent with the results found in the present study (16). 

In some studies, such as Grant et al.'s study, which investigated the level of vitamin D in patients with prostate cancer, it was shown that vitamin D supplementation can be effective in reducing the rate of prostate cancer invasion, and it seems that this may be due to from the anti-tumor effect of vitamin D or along with other anti-cancer treatments (17). In the examination of other variables that were examined in the present study, only age was related to the level of PSA. Pourmand et al., by examining the PSA serum level at different ages, reported that the PSA serum level at the age of 50 to 59 years was 1.15 ng/ml and at the age of 70 to 79 years was 1.85 ng/ml, which indicates a significant difference. There was a difference between different age groups of the study (P<0.001) (18). By examining the relationship between body mass index and PSA level, Zare et al. did not observe a relationship between PSA and body mass index, but with increasing age, the level of PSA serum level increased significantly, which is due to the occurrence of hyperplasia in old age and also a higher probability. 

The incidence of non-malignant events such as infarct in the prostate tissue was found to be consistent with the results of the present study (19). In Kim et al.'s research, the PSA serum level at the age of 21-60 years was found to be significantly lower than at the age of over 60 years (20).

 The cross-sectional nature of the study was one of the limitations of this study that the causal relationship cannot be obtained, at the same time, it was not possible to conduct a study with a higher sample size. In the present study, no significant relationship was found between PSA serum level and vitamin D level, and in the study of different variables, only a significant and direct relationship was observed between age and PSA serum level. According to the fact that 71.3% of the elderly men studied in this study had a deficiency or insufficient amount of vitamin D, It seems that health authorities can pay more attention to the adequate intake of vitamin D in the elderly, especially elderly men..
